# Is there already value in “total body PET” imaging in neurology?

**DOI:** 10.1093/bjr/tqag126

**Published:** 2026-05-29

**Authors:** Alexander Hammers

**Affiliations:** King’ College London & Guy’s and St Thomas’ PET Centre, School of Biomedical Engineering and Imaging Sciences, Faculty of Life Sciences and Medicine, King’s College London, London SE1 7EH, United Kingdom; Research Department of Biomedical Computing, School of Biomedical Engineering and Imaging Sciences, Faculty of Life Sciences and Medicine, King’s College London, London SE1 7EH, United Kingdom; Research Department of Early Life Imaging, School of Biomedical Engineering and Imaging Sciences, Faculty of Life Sciences and Medicine, King’s College London, London SE1 7EH, United Kingdom

**Keywords:** PET/CT, large axial field-of-view, brain

## Abstract

“Total Body” PET (TBP) represents a step change for all of PET, and brain imaging is no exception. Multiple benefits are already being seen. Lower injected doses benefit patients and staff and facilitate multitracer research. Shortened acquisition times for suitable radiopharmaceuticals enable better patient comfort and reduce the need for general anesthesia in children (albeit then partially negating the dose benefit). Later scanning enabled by the higher sensitivity palliates restrictions in tracer availability and allows better modeling. The large axial field-of-view enables better quantification via assessment of intravascular radioactivity concentrations. Early short-frame dynamic scanning has already enabled obtaining quantitative brain perfusion measurements for several radiopharmaceuticals. The large field-of-view allows assessment of brain-body interactions and multiorgan repercussions of disease. Despite some technical and methodological challenges remaining, TBP already offers added value in brain imaging, with exciting times ahead.

## Commentary

Prima facie, the case for a large axial field-of-view (“total body” PET, TBP) in neurological imaging is hard to make, given multiple potential disadvantages. The machines are more expensive than standard scanners. The uptake period for many radiopharmaceuticals, including the most widely used, [^18^F]FDG, cannot be shortened as it depends on biology rather than instrumentation. Similarly, scanning duration for research tracers is typically determined by tracer kinetics rather than machine performance. MRI, which is useful to have to interpret PET images in memory problems and brain tumors and essential for finding epileptogenic lesions in MRI-negative focal epilepsy patients, cannot be acquired simultaneously.

And yet, centers having adopted TBP universally use their scanners for neurological imaging, too. Why might this be? We will examine areas of added value already realized and also look at applications with potential value.

Centers have been quick to capitalize on the enormous sensitivity advantage of TBP, with very high sensitivities up to ∼170 kcps/MBq realized.^1^^,^^2^ With state-of-the-art detectors and electronics and improved resolution, image quality is generally superb. The clinical value of increased sensitivity for brain imaging is twofold.

Firstly, most centers have translated the added sensitivity at least partially into **lower injected doses—**to the point of standard imaging software now flagging potentially too low doses when loading images. Albeit the absolute risk for secondary cancers and the risk increase relative to background risk remain small, dose reduction is particularly attractive for scanning children but also in the general population, as new cancer therapies may allow much longer survival. Dose reduction is a crucial advantage for brain research, where repeat or multi-tracer studies, especially in healthy volunteers, now fit more easily within permissible ranges (eg, 10 mSv/year in the United Kingdom; https://assets.publishing.service.gov.uk/media/68077752148a9969d2394e47/Notes-for-guidance-on-the-clinical-administration-of-radiopharmaceuticals-and-use-of-sealed-radioactive-sources.pdf, p. 34, accessed August 30, 2025). The dose received by staff is also reduced per patient, and the low doses received by patient contacts and the general public after patients leave PET departments^3^ will be further reduced.

**Figure 1:**
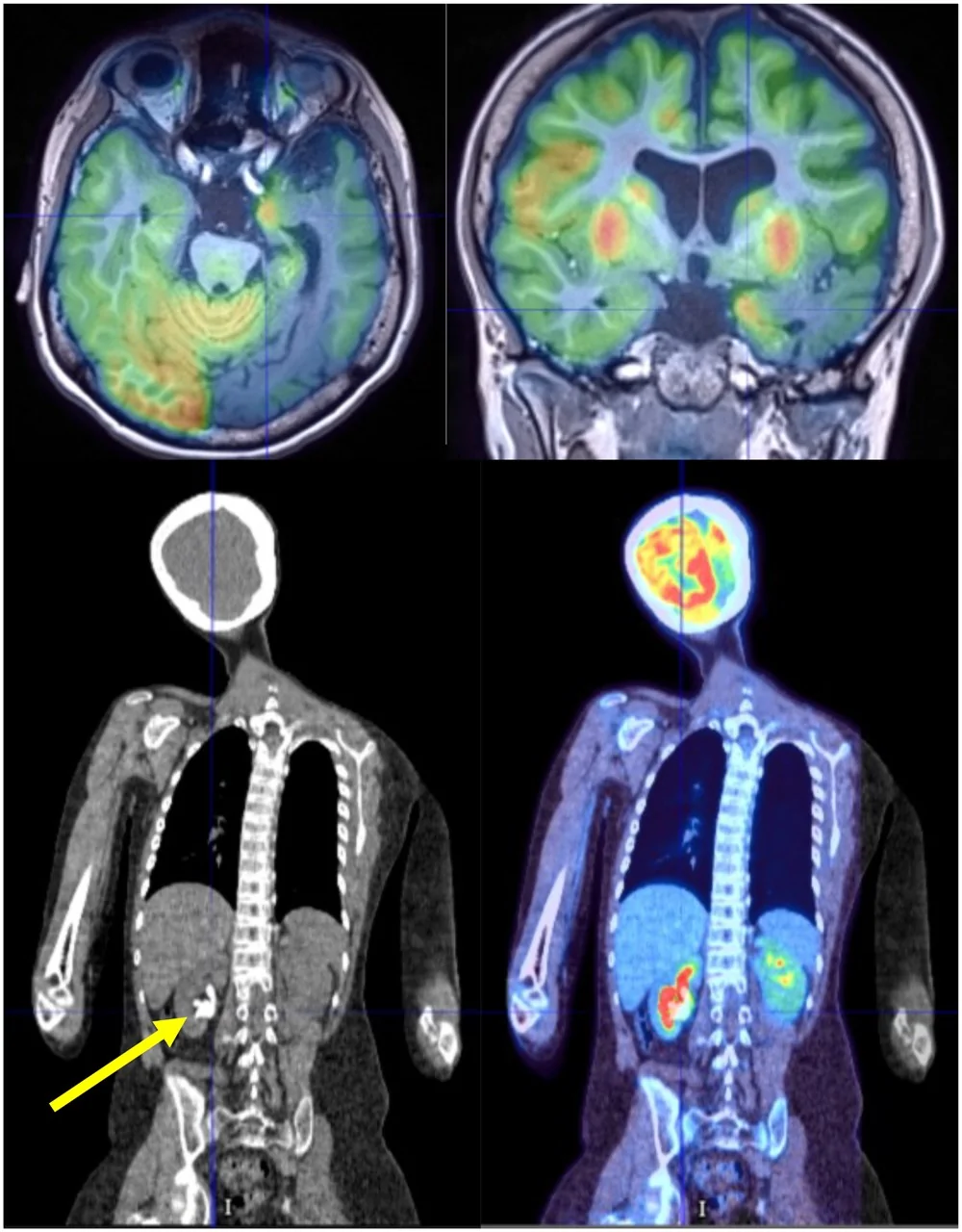
Example of useful cerebral and extracerebral information from large axial field-of-view PET—boy with learning disability assessed with [^18^F]FDG PET for epilepsy surgery. Brain PET coregistered to MRI identified the likely seizure onset zone (PET obtained 1 h postictally); body CT identified a staghorn calculus in the right kidney as a likely cause for unexplained recurrent hematuria.

Secondly, the increased sensitivity can also be used for **shortening the acquisition time**. While generally useful for neurology populations often unable to tolerate long scans, this has in particular already allowed scanning children without anesthesia (demonstrated for cancer by^4^) Children can be accustomed to the scanning environment, and emission data can then be acquired for relatively long periods, with frame durations decided post hoc depending on periods when the child has kept still. Traditionally, CTs have been acquired before emission, which in this situation would almost certainly lead to a mismatch between CT and PET for the brain (as eg, seen in Figure 1C in^4^). This is even more critical for brain than body studies, as in the brain even relatively subtle mismatches can lead to clinically relevant erroneous findings.^5^ Current innovative approaches include CT scanning only once the patient’s position during the short emission data acquisition is clear.^6^

Uptake and acquisition time are often determined by biology, for example, length of acquisition necessary to describe different kinetic behaviors or achieve sufficient lesion contrast-to-noise. However, the higher sensitivity is also an opportunity to **scan for longer** given the same initial radioactivity. For example, we have been able to acquire two consecutive long dynamic [^11^C]methionine scans in patients with brain tumors with a single synthesis. As TBP technology becomes more widespread, larger centers will likely be equipped with *n* > 1 TBP scanners, which will then be able to acquire at least nx2 dynamic acquisitions. Similarly, later scanning would also be useful for higher contrast between grey and white matter and between lesional and healthy brain tissue for FDG.^7^ Later scanning could also be useful for higher target-to-background ratios should Zr-89 labeled antibodies become available. While these are currently essentially unused in neurology, bispecific antibodies coupled with suitable chelators may cross the BBB^8^ and may become useful. Longer scan times will also facilitate modeling of binding of radiopharmaceuticals with very slow kinetics, potentially reviving use of previously “failed” tracers.

Finally, no PET image can be acquired without prior administration of a positron-emitting substance. An often-overlooked aspect is the substantially increased cost (in the United Kingdom, by about an order of magnitude) of radiopharmaceuticals due to stringent Good Manufacturing Practice requirements designed for large-batch, long shelf-life productions and, consequently, decreased variety and **availability of all radiopharmaceuticals**. By allowing lower doses and slightly longer use of radiopharmaceuticals, TBP can partially offset this hurdle and offers an opportunity for high throughput scanning from a single batch. A potential use case is scanning ahead of disease-modifying therapies for Alzheimer’s disease, where demonstration of amyloid deposition is a prerequisite and typically achieved by PET.^9^

The **extended field-of-view** and, hence, the visibility of larger areas of the body have both advantages and disadvantages. In healthy volunteers scanned with TBP, approximately 3%-6% had “useful” incidental findings (multiple personal communications), comparable to the 3%-8% of previously undiagnosed malignancies in general patient populations (reviewed in^10^). The same may happen in patients (Figure 1).

On the other hand, and similar to the situation of PET-MR, where both a nuclear medicine and a radiology report may be needed, in large departments where clinicians may be specialized in brain reading, a second person may need to read the extracerebral images—or vice versa. In addition, clinicians and departments need to decide how to deal with incidental findings in the body when the brain is the intended target organ. Ethical issues arise in particular when participants are scanned for research rather than clinical purposes.^10^

## What remains to be done?

Firstly, the dose advantage of TBP may be negated if full field-of-view CT is acquired. Efforts are underway to allow ultra-low CT dose scanning (eg,^11^) Entirely CT-free methods (eg,^12^) could be used for attenuation correction of the whole body or combined with brain-only CT.

The availability of TBP has led to calls for “whole person research.”^13^ Clinically, with the brain now systematically in the field-of-view for standard oncological imaging, there is vast potential for studying the effects of peripheral diseases such as cancer or heart failure, as well as the effects of their treatments, on the brain and leveraging this for predicting overall quality of life after treatment. Conversely, TBP should facilitate the study of interactions between brain and body, for example, regarding inflammation in relation to cardiovascular disease,^14^ psychiatric conditions such as depression,^15^ and neuroinflammation in diseases like Alzheimer’s^16^ and Huntington’s disease.^17^ More generally, TBP lends itself to the study of network interactions, with most data available for metabolism assessed with FDG.^18^ Whole-body perfusion imaging allows for fast-changing states such as anxiety induced during cardiac stress testing to be assessed.^19^ As alpha-synuclein radiopharmaceuticals become available, “brain-first” and sympathetic and parasympathetic “body-first” forms of Lewy body disorder (including Parkinson’s)^20^ could potentially be studied. Detailed assessment and validation in various diseases will be required ahead of possible translation into clinical practice.

However, many oncological studies are performed with the arms held above the head, leading to additional attenuation as well as suboptimal head immobilization. Just as motion correction became ever more important with higher field strengths allowing smaller voxel sizes in MRI, degradation of image quality through head motion that might have gone unnoticed on older systems is now clearly visible. Methods for motion minimization for dedicated brain as well as “incidental brain” oncology studies, including via head immobilization as well as post-hoc motion correction methods, should be developed and ideally standardized so they can be used by centers without their own research programs, too.

Emerging methods leveraging TBP’s high sensitivity for reconstruction of short frames, especially immediately after injection of radiotracer, may allow assessment of cerebral perfusion when combined with short (1-2 s) frames for the ubiquitous [^18^F]FDG with a distributed kinetic model^21^ or with a model-free technique as assessed for [^18^F]FDG and an additional four brain radiotracers with highly variable extraction fractions.^22^ Multi-organ quantitative perfusion assessment is highly promising for studying repercussions of disease on multiple organs (eg,^23^) Being able to routinely add brain perfusion studies to other quantitative readouts would be akin to multiple sequences acquired using MRI and could prove similarly useful.

Finally, the large field-of-view permits, for whole-blood radioactivity, image-derived functions with much greater ease than standard field-of-view scanners, as large blood pools such as the descending aorta can be sampled, minimizing partial volume effects. Other parameters such as plasma-over-blood ratio still need determining, with metabolite correction the most difficult problem.^24^ Should this prove possible at least for some tracers, research-quality quantitative data and advanced analysis methods such as (bandpass) spectral analysis^25^ could be within reach for routine clinical scanning.

## Conclusion

While some technical challenges remain to be overcome and many proof-of-concept demonstrations will require validation and implementation, there is already value in TBP in neurology, with exciting times ahead.
